# The effect of zopiclone co-administration on sertraline initial dosage optimization in pediatric major depressive disorder patients based on model-informed precision dosing

**DOI:** 10.3389/fphar.2024.1470865

**Published:** 2025-01-07

**Authors:** Xiao Chen, Ke Hu, Hao-Zhe Shi, Su-Mei He, Yang Yang, Chao-Wen Yang, Yue Zhang, Xue Tian, Ye Li, Yu-Hang Gao, Wen-Yi Xu, Cun Zhang, Dong-Dong Wang

**Affiliations:** ^1^ School of Nursing, Xuzhou Medical University, Xuzhou, Jiangsu, China; ^2^ Jiangsu Key Laboratory of New Drug Research and Clinical Pharmacy and School of Pharmacy, Xuzhou Medical University, Xuzhou, Jiangsu, China; ^3^ Department of Pharmacy, Suzhou Research Center of Medical School, Suzhou Hospital, Affiliated Hospital of Medical School, Nanjing University, Suzhou, Jiangsu, China; ^4^ Department of Pharmacy, The Affiliated Changzhou Children’s Hospital of Nantong University, Changzhou, Jiangsu, China; ^5^ Department of Pharmacy, Xuzhou Oriental Hospital Affiliated to Xuzhou Medical University, Xuzhou, Jiangsu, China

**Keywords:** zopiclone, sertraline, initial dosage optimization, pediatric major depressive disorder, model-informed precision dosing, drug-drug interaction

## Abstract

**Objective:**

The present study aims to explore the initial dosage optimization of sertraline in pediatric major depressive disorder (MDD) patients based on model-informed precision dosing (MIPD).

**Methods:**

A total of 111 pediatric MDD patients treated with sertraline were included for analysis using MIPD. Sertraline concentration levels, physiological and biochemical indexes of pediatric MDD patients, combined drug information were included in the construction of model.

**Results:**

Weight and zopiclone co-administration influenced sertraline clearance in pediatric MDD patients. With the same weight, the sertraline clearance rates were 0.453:1 in patients with, or without zopiclone, respectively. Furthermore, without zopiclone, for once-daily sertraline scheme, the dosages of 4.0, and 3.0 mg/kg/day were suggested for pediatric MDD patients with body weight of 30–38.5, and 38.5–80 kg, respectively; for twice-daily sertraline scheme, the dosage of 2.0 mg/kg/day was suggested for pediatric MDD patients with body weight of 30–80 kg. With zopiclone, for once-daily sertraline scheme, the dosage of 1.0 mg/kg/day was suggested for pediatric MDD patients with body weight of 30–80 kg; for twice-daily sertraline scheme, the dosage of 0.5 mg/kg/day was suggested for pediatric MDD patients with body weight of 30–80 kg.

**Conclusion:**

This study first explored the initial dosage optimization of sertraline in pediatric MDD patients based on MIPD, and recommended the optimal sertraline initial dosage in pediatric MDD patients based on zopiclone co-administration.

## 1 Introduction

Major depressive disorder (MDD) is one of the most common mental illnesses, with a prevalence of 3.02% (Smith, 2014). The long course of MDD requires a long-term standardized treatment, bringing heavy economic and mental double burden to society and patient’s family, and it is predicted that by 2030 MDD will become the world’s second largest disease burden (Mathers and Loncar, 2006)**.** In recent years, it has been found that the prevalence of MDD in children has increased year by year Liu et al. (2023). The duration of childhood MDD is longer than that of adult MDD and the younger the age of first onset, the higher the recurrence rate and suicide risk, which seriously affects interpersonal communication of children (Alaie et al., 2021).

Medication is one of the main ways to treat MDD in children, and studies have confirmed that sertraline is safe and effective in the treatment of pediatric MDD patients (Park and Goodyer, 2000; Rushton, 2004). Sertraline is a powerful class of serotonin reuptake inhibitors (SSRIs) that are clinically used to treat different types of MDD. Sertraline metabolism is mediated by several cytochrome (CYP) P450 enzymes, mainly include CYP3A4, CYP2B6, CYP2D6, CYP2C9, CYP2C19 (Obach et al., 2005), and some drugs who may inhibit or activate these enzymes that have a profound effect on sertraline metabolism may have potential drug-drug interaction with sertraline during clinical combination administration, which may affect the concentration and dosage of sertraline in pediatric MDD patients.

The pharmacokinetics of sertraline has been evaluated in adults and demonstrates extensive variability (Poweleit et al., 2023). Sertraline is slowly absorbed, reaching maximum concentrations in plasma at 4–8 h after oral administration, absolute bioavailability is estimated to be >44% (DeVane et al., 2002). In addition, co-administration of sertraline with food increases peak plasma concentration by approximately 25% and decreases time to peak concentration from 8–5.5 h, with a net marginal increase in area under the concentration-time curve (DeVane et al., 2002). In pediatric patients, sertraline exposure is 22% lower after adjusting plasma concentrations for weight compared to adults (Alderman et al., 1998). The serum protein binding rate of sertraline is approximately 98%–99% (Kristensen et al., 1998), and sertraline concentration changes may be connection with drug efficacy and side effects, and quantitatively interpreting and predicting sertraline concentration changes in the body according to exposure to sertraline will be important (Jang and Jeong, 2024). Inter-individual variation of sertraline pharmacokinetics is sizeable, and several physiological, biochemical, genetic and drug combination parts have been implicated as potential factors involved (Tini et al., 2022). However, the discovery of effective covariates related to the interpretation of sertraline pharmacokinetics diversity is still insufficient and continued exploration is required (Jang and Jeong, 2024). Additionally, due to study in pediatric patients is limited, the pharmacokinetics of sertraline in pediatric populations and the influence of drug-drug interaction in this specific group need to be urgently studied (Poweleit et al., 2023).

Model-informed precision dosing (MIPD) integrates information about patients, drugs, and diseases through mathematical modeling and simulation techniques to provide a basis for patient precision medicine. Compared with empirical medicine, MIPD is a new method to formulate drug administration plan based on the characteristics of patients’ physiology, pathology, heredity and disease, which can improve the safety, effectiveness, economy and compliance of drug therapy, in addition, MIPD can identify potential drug-drug interaction and optimize initial dosage (Hu et al., 2023).

Therefore, this study aims to explore the precise administration of sertraline in pediatric MDD patients using MIPD, identifying drug combination that interact with sertraline in pediatric MDD patients, guiding individual medication of sertraline in pediatric MDD patients, and reducing the treatment failure of pediatric MDD patients caused by insufficient or excessive exposure of sertraline due to drug-drug interaction.

## 2 Methods

### 2.1 Data collection

Pediatric MDD patients treated by sertraline from *Xuzhou Oriental Hospital Affiliated to Xuzhou Medical University* (Ethical approval number: 20220725011) between September 2021 and January 2024 were collected, retrospectively. Sertraline concentrations of pediatric MDD patients were obtained from therapeutic drug monitoring (TDM) database.

### 2.2 Modeling

We chose population pharmacokinetics (PPK) as the MIPD model, which was built via the non-linear mixed effect modeling (NONMEM) software (Edition 7.4.1, ICON Development Solutions, Ellicott City, MD, United States). Given trough concentrations, we researched the absorption phase with a one-compartment model. Sparse trough concentrations monitored by TDM could provide insufficient information, and a lag time of absorption and bioavailability (F) could not be evaluated. Therefore, our pharmacokinetic parameters mainly included apparent oral clearance (CL/F), volume of distribution (V/F), and Ka, the absorption rate constant of the model. Furtherly, we attempted to estimate Ka at 0.01/h, 0.4/h, 0.5/h, 0.6/h, 1.0/h, and 2.0/h.

Formula 1 was used to assess the inter-individual variability:

$$A_{i}=\text{TV}\left(A\right)\times \exp\left(\eta_{i}\right)$$ (1)

A_i_ was individual parameter. TV (A) was typical individual parameter. η_i_ was the inter-individual variability and represented symmetrical distribution, which was random term with zero mean and variance omega^∧^2 (ω^2^).

Formula 2 was used to assess the random residual variability:

$$B_{i}=C_{i}+C_{i*}\varepsilon_{1}+\varepsilon_{2}$$ (2)

B_i_ was observed concentration. C_i_ was individual predicted concentration. ε_1_ was the proportional residual variability and ε_2_ was the additive residual variability. ε_1_ and ε_2_ represented symmetrical distribution, which were random term with zero mean and variance sigma^∧^2 (σ^2^).

Formula 3 was used to assess the relationship of pharmacokinetic parameters with weight:

$$D_{i}=D_{\text{std}}\times {\left(E_{i}/E_{\text{std}}\right)}^{F}$$ (3)

D_i_ was i-th individual parameter. E_i_ was i-th individual weight. E_std_ was standard weight of 70 kg and D_std_ was typical individual parameter, whose weight was E_std_. F was the allometric coefficient: 0.75 for the CL/F and 1 for the V/F (Anderson and Holford, 2008).

Formulas 4, 5 were used to assess the pharmacokinetic parameters between continuous covariates or categorical covariates, respectively:

$$G_{i}=\text{TV}\left(G\right)\times {\left({\text{Cov}}_{i}\;/{\text{Cov}}_{m}\right)}^{\theta }$$ (4)

$$G_{i}=\text{TV}\left(G\right)\times \left(1+\theta \times {\text{Cov}}_{i}\right)$$ (5)

G_i_ was individual parameter. TV (G) was typical individual parameter. θ was parameter to be estimated. Cov_i_ was covariate of the i-th individual. Cov_m_ was population median for the covariate.

The covariate model of PPK was carried out by a stepwise way, where the objective function value (OFV) changes were selected as the covariate inclusion criteria. The decrease of OFV > 3.84 (*P* < 0.05) was deemed to the inclusion standard and increase of OFV > 6.63 (*P* < 0.01) was deemed to the exclusion standard.

### 2.3 Model validation

The final model was validated using visualization and the median and 2.5–97.5 th percentiles of bootstrap (n = 1,000) were used for comparing with final model parameters.

### 2.4 Simulation

Monte Carlo simulation was used for forecasting sertraline initial dosage optimization, where the recommended therapeutic window for sertraline in pediatric MDD patients was 10–150 ng/mL (Poweleit et al., 2023). We thoroughly investigated the possible interactions with other drugs that the patients might be using, included alprazolam tablet, aripiprazole tablet, benzoxol hydrochloride tablet, buspirone hydrochloride tablet, clonazepam tablet, diazepam injection, haloperidol injection, levetiracetam tablet, lithium carbonate extended-release tablet, lorazepam tablet, olanzapine tablet, oxazepam tablet, perphenazine tablet, piropilone hydrochloride tablet, pregabalin capsule, propranolol hydrochloride tablet, quetiapine fumarate tablet, sodium valproate sustained-release tablet, sodium valproate tablet, zopiclone tablet. Furtherly, the influential covariates from the PPK model were include in the simulations.

## 3 Results

### 3.1 Patient information

A total of 111 pediatric MDD patients treated with sertraline were included for analysis, where 23 boys and 88 girls, whose ages were from 11.04–16.00 years old, weights were from 35.00–117.00 kg. Demographic data of pediatric MDD patients and drug combination were shown in Tables 1, 2, respectively.

**TABLE 1:** Demographic data of patients (n = 111).

Characteristic	Number or mean ± SD	Median (range)
Gender (boys/girls)	23/88	—
Age (years)	14.38 ± 0.86	14.17 (11.04–16.00)
Weight (kg)	56.58 ± 15.01	52.50 (35.00–117.00)
Albumin (g/L)	41.99 ± 2.37	41.90 (35.60–50.60)
Globulin (g/L)	25.20 ± 2.37	25.05 (19.70–32.40)
Alanine transaminase (IU/L)	37.65 ± 44.95	20.00 (1.00–267.00)
Aspartate transaminase (IU/L)	28.34 ± 21.78	20.00 (12.00–147.00)
Creatinine (μmol/L)	49.68 ± 9.24	50.00 (30.00–73.00)
Urea (mmol/L)	4.03 ± 1.19	3.84 (1.59–8.06)
Total protein (g/L)	67.19 ± 3.65	66.75 (59.70–82.80)
Total cholesterol (mmol/L)	4.42 ± 0.86	4.30 (2.70–6.78)
Triglyceride (mmol/L)	1.37 ± 0.77	1.12 (0.38–4.79)
Direct bilirubin (μmol/L)	2.02 ± 1.07	1.90 (0.20–7.00)
Total bilibrubin (μmol/L)	6.69 ± 3.11	5.90 (1.70–21.10)
Hematocrit (%)	37.36 ± 3.89	36.95 (27.40–48.80)
Hemoglobin (g/L)	123.32 ± 15.02	122.00 (82.00–163.00)
Mean corpuscular hemoglobin (pg)	28.80 ± 2.47	29.35 (20.60–33.40)
Mean corpuscular hemoglobin concentration (g/L)	329.61 ± 11.72	330.00 (293.00–363.00)

**TABLE 2:** Drug combination in pediatric major depressive disorder patients (n = 111).

Drug	Category	N	Drug	Category	N
Alprazolam tablet	0	93	Olanzapine tablet	0	69
	1	18		1	42
Aripiprazole tablet	0	101	Oxazepam tablet	0	106
	1	10		1	5
Benzoxol hydrochloride tablet	0	109	Perphenazine tablet	0	109
	1	2		1	2
Buspirone hydrochloride tablet	0	109	Piropilone hydrochloride tablet	0	110
	1	2		1	1
Clonazepam tablet	0	57	Pregabalin capsule	0	110
	1	54		1	1
Diazepam injection	0	109	Propranolol hydrochloride tablet	0	109
	1	2		1	2
Haloperidol injection	0	109	Quetiapine fumarate tablet	0	59
	1	2		1	52
Levetiracetam tablet	0	110	Sodium valproate sustained-release tablet	0	109
	1	1		1	2
Lithium carbonate extended-release tablet	0	86	Sodium valproate tablet	0	108
	1	25		1	3
Lorazepam tablet	0	107	Zopiclone tablet	0	108
	1	4		1	3

Category, 0: without drug, 1: with drug; N: number of patients.

### 3.2 Modeling

Through evaluation we found that when Ka were at 0.01/h, 0.4/h, 0.5/h, 0.6/h, 1.0/h, and 2.0/h, the OFV were 1,138.021, 1,104.104, 1,104.200, 1,104.208, 1,104.129, 1,104.020, respectively. Combined with the fact that Ka was fixed at 0.5/h in previous literature (Poweleit et al., 2023), we finally fixed Ka at 0.5 in our study. Weight was fixed via allometric exponent (Anderson and Holford, 2008). In addition, it was found that only zopiclone was included in the final model and had significant interaction with sertraline, as shown in Supplementary Table S1 for the research process. Thus, weight and zopiclone co-administration influenced sertraline clearance in pediatric MDD patients. The final PPK model of sertraline in pediatric MDD patients were as follow, which were shown in Formulas 6, 7:

$$\text{CL}/F=74\times {\left(\text{weight}/70\right)}^{0.75}\times \left(1-0.547\times \text{ZOP}\right)$$ (6)

$$V/F=874\times \left(\text{weight }/70\right)$$ (7)

CL/F was apparent oral clearance and V/F was apparent volume of distribution. ZOP was zopiclone, when pediatric MDD patients took zopiclone, ZOP was 1, otherwise ZOP was 0.

### 3.3 Evaluation

**FIGURE 1:**
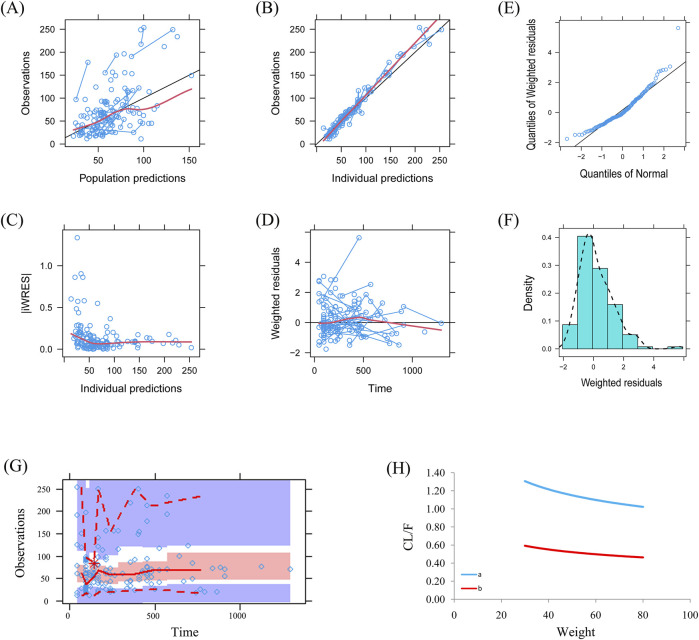
Model evaluation. **(A)** Observations vs. population predictions. **(B)** Observations vs. individual predictions. **(C)** absolute value of weighted residuals of individual (│iWRES│) vs. individual predictions. **(D)** Weighted residuals vs. time. **(E)** Quantilies of weighted residuals vs. quantilies of normal. **(F)** Density vs. weighted residuals. **(G)** Visual predictive check (VPC) of model. **(H)** Sertraline clearance, **(A)** without zopiclone, **(B)** with zopiclone.

**FIGURE 2:**
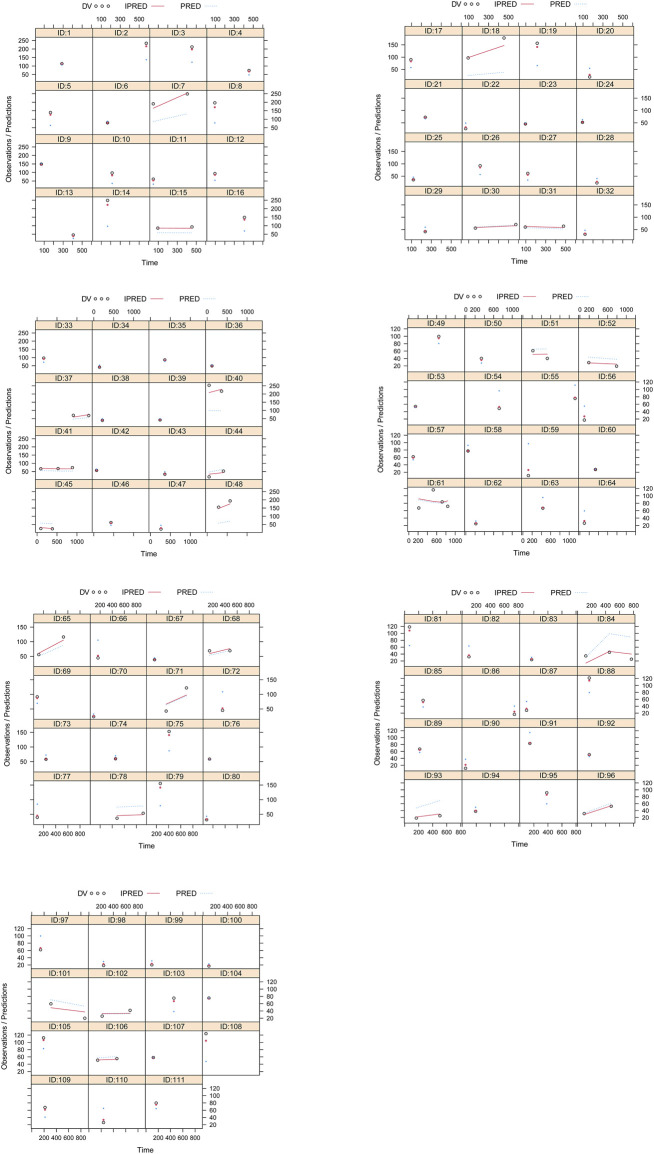
Individual plots. ID: patient ID number. DV: measured concentration value. IPRED, individual predictive value. PRED, population predictive value.

Visual evaluation index and VPC of model were shown in Figures 1A–G, respectively, showing the sertraline concentrations were well predicted by the final PPK model. Figure 1H showed that with the same weight, the sertraline clearance rates were 54.7% lower in patients taking zopiclone than those not taking zopiclone. Individual plots were shown in Figure 2, showing the final model may predict the sertraline concentrations of pediatric MDD patients well at the individual level. Table 3 showed the parameter estimates of the final model and bootstrap validation, whose biases were all less than ±3%, showing the final model was accurate and reliable.

**TABLE 3:** Parameter estimates of final model and bootstrap validation (n = 1,000).

Parameter	Estimate	SE (%)	Bootstrap		Bias (%)
			Median	95% Confidence interval	
CL/F (L/h)	74	12.1	74	[43, 91]	0
V/F (L)	874	36.6	886	[215, 2013]	1.37
Ka (h^-1^)	0.5 (fixed)	--	--	--	--
Allometric exponent of weight on CL/F (L/h)	0.75 (fixed)	--	--	--	--
Allometric exponent of weight on V/F (L)	1 (fixed)	--	--	--	--
θ_ZOP_	−0.547	19.0	−0.550	[−0.727, −0.245]	0.55
ω_CL/F_	0.391	11.4	0.383	[0.266, 0.481]	−2.05
σ_1_	0.159	22.4	0.157	[0.076, 0.223]	−1.26
σ_2_	9.803	18.0	9.534	[3.173, 12.500]	−2.74

95% confidential interval was displayed as the 2.5th, 97.5th percentile of bootstrap estimates. CL/F, apparent oral clearance (L/h); V/F, apparent volume of distribution (L); Ka, absorption rate constant (h^−1^); θ_ZOP_, was the coefficient of zopiclone; ω_CL/F_, inter-individual variability of CL/F; σ_1_, residual variability, proportional error; σ_2_, residual variability, additive error; Bias, prediction error, Bias = (Median-Estimate)/Estimate × 100%.

### 3.4 Simulation

On the basis of whether zopiclone was used in combination, and a once-daily or a twice-daily sertraline administration, we simulated four different situations: (1) Once-daily sertraline administration dosage without zopiclone, (2) Twice-daily sertraline administration dosage without zopiclone, (3) Once-daily sertraline administration dosage with zopiclone, (4) Twice-daily sertraline administration dosages with zopiclone, where each situation included 1,000 virtual pediatric MDD patients, six dosages (0.5, 1.0, 2.0, 3.0, 4.0, 5.0 mg/kg/day) for six weight groups (30, 40, 50, 60, 70, 80 kg), respectively. The twice-daily sertraline administration dosage was split evenly into two dosages a day. The evaluation criterion was the probability to achieve target concentrations.

**FIGURE 3:**
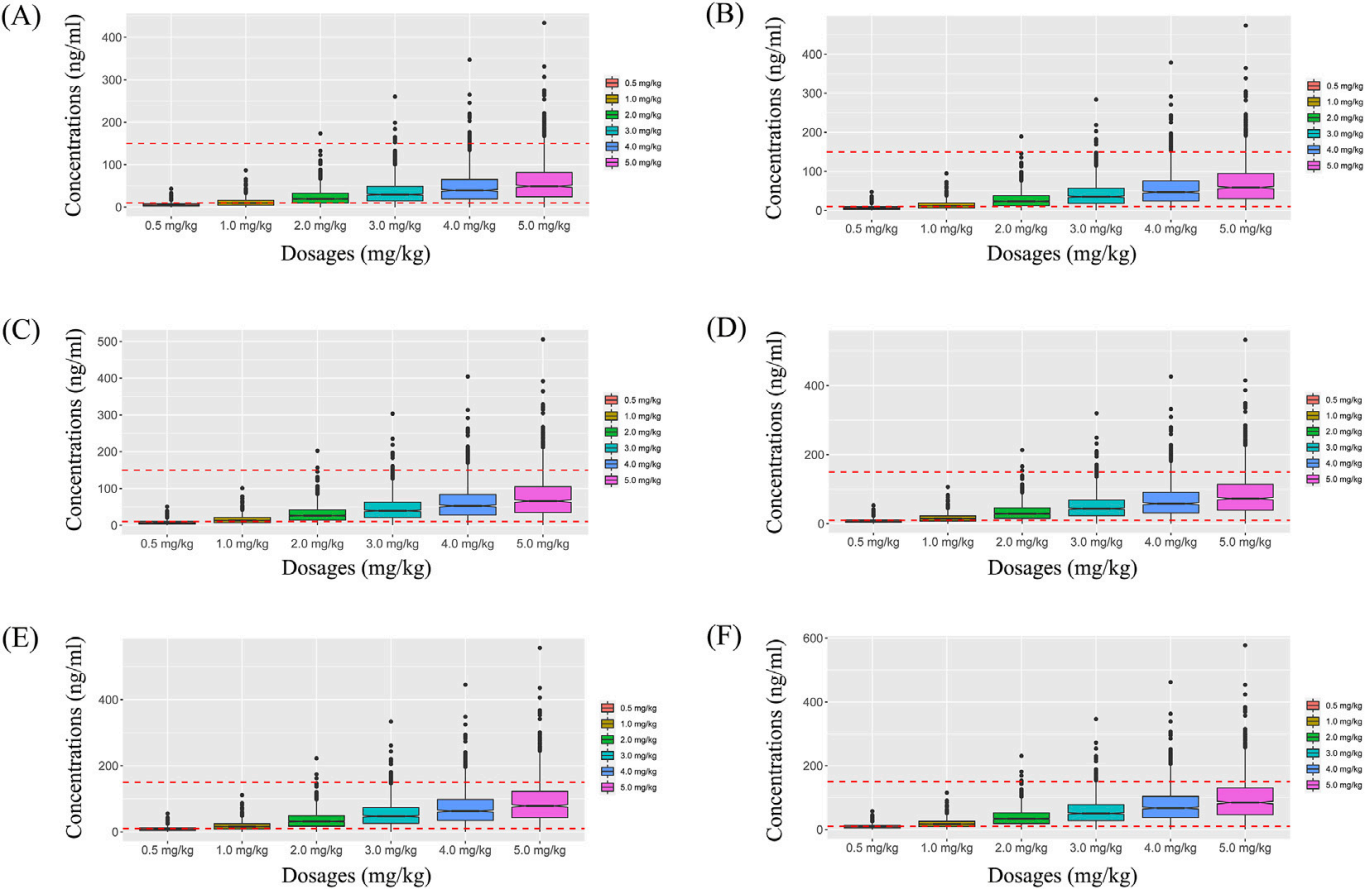
Simulated sertraline concentrations from once-daily sertraline administration dosage without zopiclone. **(A)** Pediatric MDD patients with 30 kg, **(B)** Pediatric MDD patients with 40 kg, **(C)** Pediatric MDD patients with 50 kg, **(D)** Pediatric MDD patients with 60 kg, **(E)** Pediatric MDD patients with 70 kg, **(F)** Pediatric MDD patients with 80 kg.

**FIGURE 4:**
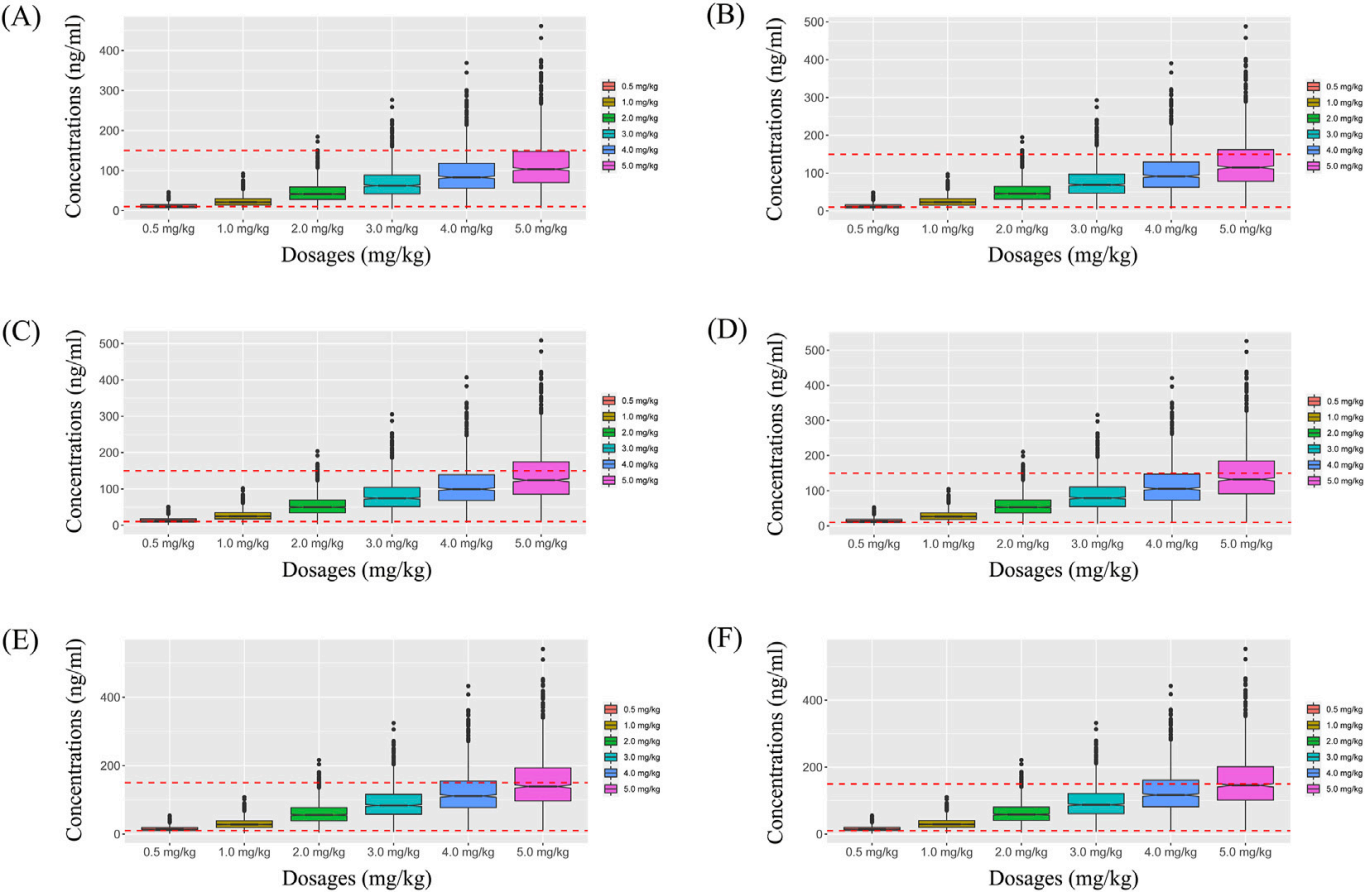
Simulated sertraline concentrations from twice-daily sertraline administration dosage without zopiclone. **(A)** Pediatric MDD patients with 30 kg, **(B)** Pediatric MDD patients with 40 kg, **(C)** Pediatric MDD patients with 50 kg, **(D)** Pediatric MDD patients with 60 kg, **(E)** Pediatric MDD patients with 70 kg, **(F)** Pediatric MDD patients with 80 kg.

**FIGURE 5:**
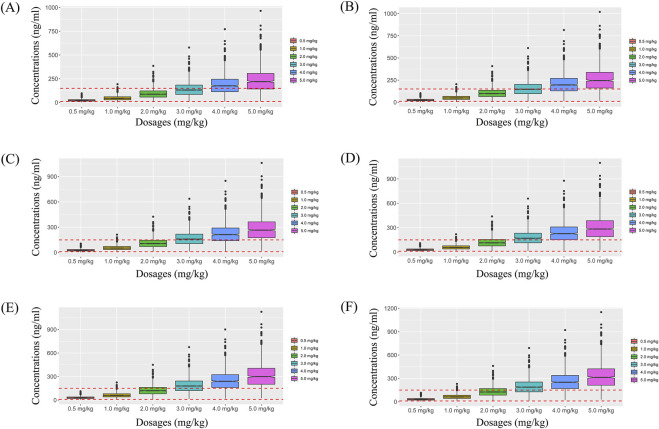
Simulated sertraline concentrations from once-daily sertraline administration dosage with zopiclone. **(A)** Pediatric MDD patients with 30 kg, **(B)** Pediatric MDD patients with 40 kg, **(C)** Pediatric MDD patients with 50 kg, **(D)** Pediatric MDD patients with 60 kg, **(E)** Pediatric MDD patients with 70 kg, **(F)** Pediatric MDD patients with 80 kg.

**FIGURE 6:**
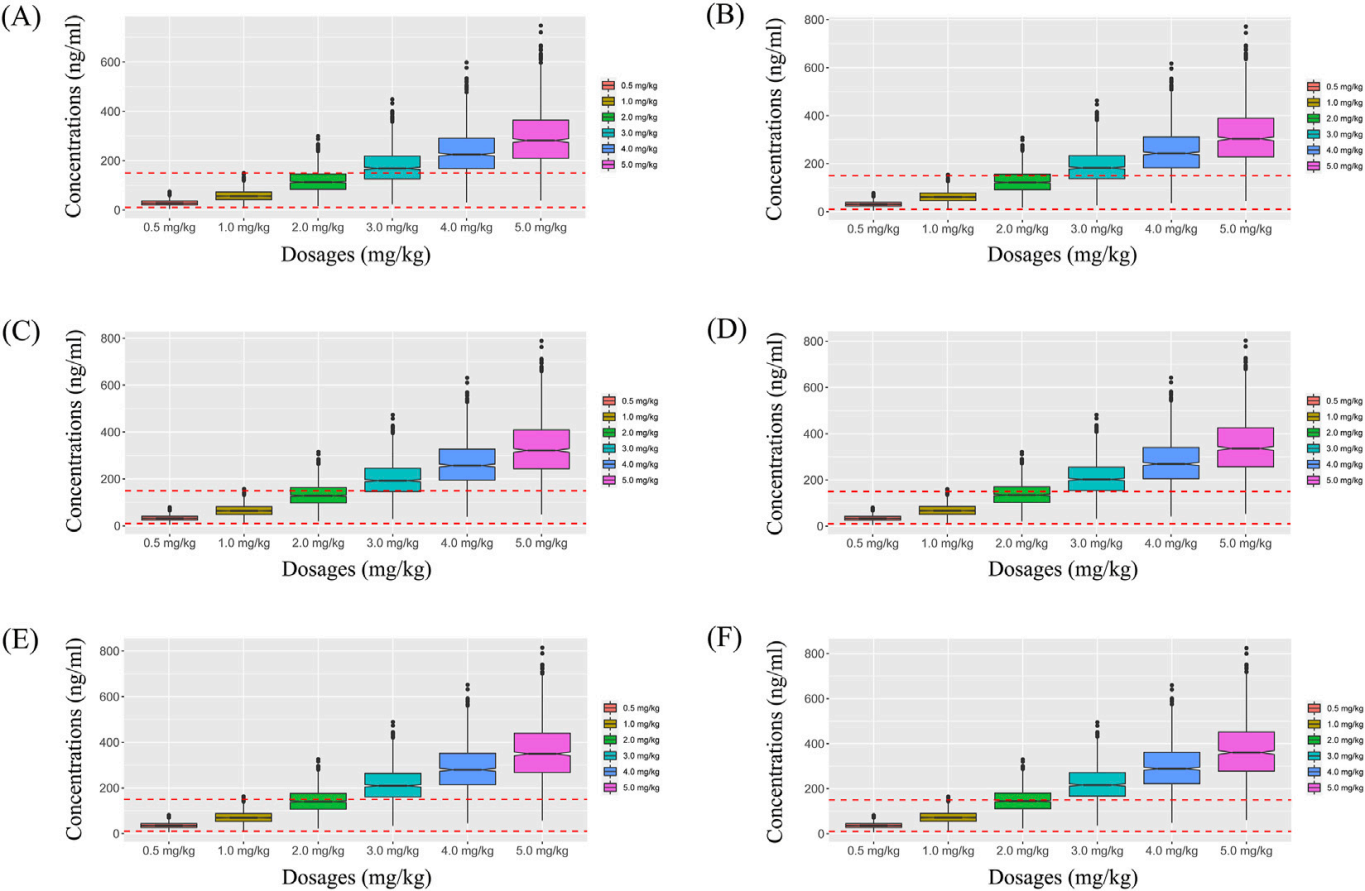
Simulated sertraline concentrations from twice-daily sertraline administration dosage with zopiclone. **(A)** Pediatric MDD patients with 30 kg, **(B)** Pediatric MDD patients with 40 kg, **(C)** Pediatric MDD patients with 50 kg, **(D)** Pediatric MDD patients with 60 kg, **(E)** Pediatric MDD patients with 70 kg, **(F)** Pediatric MDD patients with 80 kg.

**FIGURE 7:**
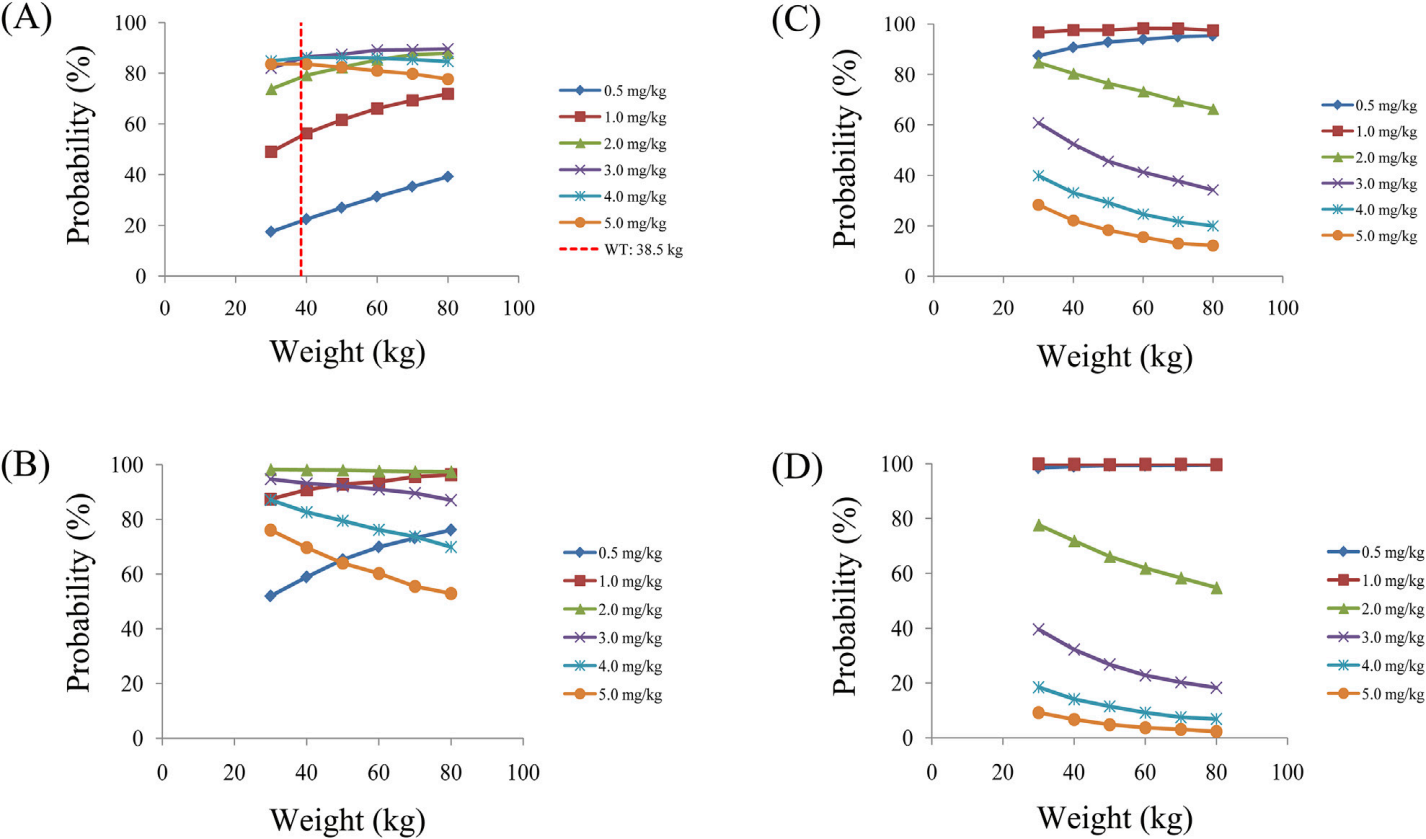
Probabilities to achieve therapeutic window. **(A)** Once-daily sertraline administration dosage without zopiclone, **(B)** Twice-daily sertraline administration dosage without zopiclone, **(C)** Once-daily sertraline administration dosage with zopiclone, **(D)** Twice-daily sertraline administration dosages with zopiclone.

Simulated sertraline concentrations from once-daily sertraline administration dosage without zopiclone, twice-daily sertraline administration dosage without zopiclone, once-daily sertraline administration dosage with zopiclone, twice-daily sertraline administration dosages with zopiclone were shown in Figures 3–6, respectively. The probabilities to achieve the target concentrations were shown in Figure 7, where Figures 7A–D were once-daily sertraline administration dosage without zopiclone, twice-daily sertraline administration dosage without zopiclone, once-daily sertraline administration dosage with zopiclone, twice-daily sertraline administration dosages with zopiclone, respectively. The recommended optimal sertraline initial dosage in pediatric MDD patients were shown in Table 4.

**TABLE 4:** Initial dosage recommendation of sertraline in pediatric major depressive disorder patients without or with zopiclone.

Without zopiclone			With zopiclone		
Once a day			Once a day		
Body weight (kg)	Dose (mg/kg/day)	Probability to achieve the target concentrations (%)	Body weight (kg)	Dose (mg/kg/day)	Probability to achieve the target concentrations (%)
30–38.5	4.0	84.9–86.3	30–80	1.0	96.7–98.3
38.5–80	3.0	86.3–89.7			

Split evenly into two doses a day			Split evenly into two doses a day		
Body weight (kg)	Dose (mg/kg/day)	Probability to achieve the target concentrations (%)	Body weight (kg)	Dose (mg/kg/day)	Probability to achieve the target concentrations (%)
30–80	2.0	97.4–98.2	30–80	0.5	98.5–99.6

For example, Figures 3A–F were simulated sertraline concentrations from once-daily sertraline administration dosage without zopiclone in pediatric MDD patients with 30 kg, 40 kg, 50 kg, 60 kg, 70 kg and 80 kg, respectively. In Figure 3A, different color “weight boxes” represented concentrations at different simulated dosages, among which the part between the two red dashed lines represented the concentration points reaching the therapeutic window. The evaluation criterion was the probability to achieve target concentrations. In this way, in pediatric MDD patients with 30 kg, simulated 0.5 mg/kg, 1.0 mg/kg, 2.0 mg/kg, 3.0 mg/kg, 4.0 mg/kg, 5.0 mg/kg sertraline dosages corresponded to six probability values of reaching the treatment window, respectively. By analogy, in pediatric MDD patients with 40 kg, 50 kg, 60 kg, 70 kg and 80 kg, simulated 0.5 mg/kg, 1.0 mg/kg, 2.0 mg/kg, 3.0 mg/kg, 4.0 mg/kg, 5.0 mg/kg sertraline dosages, the simulated dosages would correspond to its own probability values of reaching the treatment window. Finally, when the simulated sertraline dosage was 0.5 mg/kg, pediatric MDD patients with 30 kg, 40 kg, 50 kg, 60 kg, 70 kg and 80 kg each has a probability value of reaching the treatment window. Furtherly, we made probability figure, the dark blue line with slanted square was determined by simulated 0.5 mg/kg sertraline dosage, weight as horizontal coordinate and probability value as vertical coordinate. In a similar way, the probability lines of 1.0 mg/kg, 2.0 mg/kg, 3.0 mg/kg, 4.0 mg/kg, 5.0 mg/kg sertraline dosages were done. The simulated dosage with the highest probability in the same body weight range was the optimized dosage, which was shown in Figure 7A, the dosages of 4.0, and 3.0 mg/kg/day were optimization for pediatric MDD patients with body weight of 30–38.5, and 38.5–80 kg, respectively. Similarly, Figures 7B–D were the exploration way of optimized dosage for twice-daily sertraline administration dosage without zopiclone, once-daily sertraline administration dosage with zopiclone, twice-daily sertraline administration dosages with zopiclone, respectively.

In a word, without zopiclone, for once-daily sertraline scheme, the dosages of 4.0, and 3.0 mg/kg/day were suggested for pediatric MDD patients with body weight of 30–38.5, and 38.5–80 kg, respectively, and simultaneously, the probabilities to achieve the target concentrations for the dosages of 4.0, 3.0 mg/kg/day were 84.9%–86.3%, 86.3%–89.7%, respectively; for twice-daily sertraline scheme, the dosage of 2.0 mg/kg/day was suggested for pediatric MDD patients with body weight of 30–80 kg, and simultaneously, the probability to achieve the target concentrations for the dosage of 2.0 mg/kg/day was 97.4%–98.2%. With zopiclone, for once-daily sertraline scheme, the dosage of 1.0 mg/kg/day was suggested for pediatric MDD patients with body weight of 30–80 kg, and simultaneously, the probability to achieve the target concentrations for the dosage of 1.0 mg/kg/day was 96.7%–98.3%; for twice-daily sertraline scheme, the dosage of 0.5 mg/kg/day was suggested for pediatric MDD patients with body weight of 30–80 kg, and simultaneously, the probability to achieve the target concentrations for the dosage of 0.5 mg/kg/day was 98.5%–99.6%.

In other words, how the findings can be applied in real-world settings? When pediatric MDD patients do not take zopiclone at the same time, and if the once-daily sertraline scheme is clinically intended to be adopted, pediatric MDD patients with body weight of 30–38.5, and 38.5–80 kg will be recommended to take 4.0, and 3.0 mg/kg/day sertraline, respectively; If the twice-daily sertraline scheme is clinically intended to be adopted, pediatric MDD patients with body weight of 30–80 kg will be recommended to take 2.0 mg/kg/day sertraline. On the other hand, when pediatric MDD patients take zopiclone at the same time, and if the once-daily sertraline scheme is clinically intended to be adopted, pediatric MDD patients with body weight of 30–80 kg will be recommended to take 1.0 mg/kg/day sertraline; If the twice-daily sertraline scheme is clinically intended to be adopted, pediatric MDD patients with body weight of 30–80 kg will be recommended to take 0.5 mg/kg/day sertraline.

## 4 Discussion

Sertraline has narrow therapeutic window, large pharmacokinetic differences between individuals and within individuals, and drug concentration is closely related to efficacy and adverse reactions, where TDM is often used to adjust the dosage of sertraline in the clinical treatment of MDD patients (Li et al., 2013; Alhadab and Brundage, 2020; Poweleit et al., 2023; Stoiljkovic et al., 2023; Zhang et al., 2024). The recommended therapeutic window for sertraline is 10–150 ng/mL (Poweleit et al., 2023), which provides strong support for individualized administration of sertraline in patients with MDD. In diagnosis and treatment practice, the next dosage of sertraline can be adjusted according to TDM feedback to achieve individual drug administration need. However, for the initial dosage, there is no available TDM value as a reference to formulate an accurate initial sertraline administration regimen. At the same time, there may be potential drug-drug interaction in the course of clinical drug combination, which affects the concentration and dosage of sertraline in pediatric MDD patients.

In this study, we explored the individualized and precise administration of sertraline in pediatric MDD patients through MIPD, and formulated the optimal initial administration regimen of sertraline in pediatric MDD patients based on drug-drug interaction. A total of 111 pediatric MDD patients taking sertraline were included in the study for modeling analysis of MIPD. There was a demographic makeup imbalance, with a notable disparity in the number of female participants compared to male participants and the female to male ratio was 79.3% vs*.* 20.7%. However, this problem was unavoidable in population pharmacokinetic studies and it had no substantial impact on our research of individualized drug administration. Many studies of similar sex ratio had been reported. For example, in the study of population pharmacokinetics of vancomycin in intensive care patients with the time-varying status of temporary mechanical circulatory support or continuous renal replacement therapy, the male to female ratio was 96% vs*.* 4% (Tsai et al., 2024). In the study of an integrated population pharmacokinetic model of febuxostat in pediatric patients with hyperuricemia including gout and adult population of healthy subjects and patients with renal dysfunction, the male to female ratio was 79.3% vs*.* 20.7% (Iwama et al., 2024). In the study of population pharmacokinetics and dose evaluations of linezolid in the treatment of multidrug-resistant tuberculosis, the male to female ratio was 74.4% vs*.* 25.6% (Zhang et al., 2022). In the study of population pharmacokinetics of unfractionated heparin and multivariable analysis of activated clotting time in patients undergoing radiofrequency ablation of atrial fibrillation, the male to female ratio was 75.9% vs*.* 24.1% (Konecki et al., 2024).

In the final model, we found that weight and zopiclone co-administration influenced sertraline clearance in pediatric MDD patients. With the same weight, the sertraline clearance rates were 0.453:1 in patients with, or without zopiclone, respectively. The influence of body weight on drug clearance in children had been reported in many studies (Ni et al., 2013; Hao et al., 2018; Chen et al., 2022). In addition, zopiclone was a commonly prescribed nonbenzodiazepine hypnotic drug and it was used as a short-term treatment strategy to improve sleep in a number of psychiatric disorders, where CYP3A4 was the major enzyme involved in zopiclone metabolism (Becquemont et al., 1999). The effect of zopiclone co-administration on sertraline clearance was probably from competing with sertraline for the metabolic enzyme CYP3A4.

Monte Carlo simulation was a computational method to simulate complex systems by random sampling and probability statistics, which was widely used in clinical individualized drug delivery research. For example, Xie *et al* reported managing delayed or missed pregabalin doses in patients with focal epilepsy: a Monte Carlo simulation study (Xie et al., 2024). Rao *et al* reported optimal dosing regimen of biapenem based on population pharmacokinetic/pharmacodynamic modelling and Monte Carlo simulation in patients with febrile neutropenia and haematological malignancies (Rao et al., 2023). Zheng *et al* reported population pharmacokinetic modeling using polymyxin B free plasma concentrations from published reports and evaluation of dosage regimens based on Monte Carlo simulation in critically ill patients (Zheng et al., 2023). Perez *et al* reported population pharmacokinetics of isavuconazole in critical care patients with COVID-19-associated pulmonary aspergillosis and Monte Carlo simulations of high off-label doses (Perez et al., 2023). Furthermore, we simulated different dosages using Monte Carlo simulation to optimize the optimal initial administration regimen of sertraline. Without zopiclone, for once-daily sertraline scheme, the dosages of 4.0, and 3.0 mg/kg/day were suggested for pediatric MDD patients with body weight of 30–38.5, and 38.5–80 kg, respectively; for twice-daily sertraline scheme, the dosage of 2.0 mg/kg/day was suggested for pediatric MDD patients with body weight of 30–80 kg. With zopiclone, for once-daily sertraline scheme, the dosage of 1.0 mg/kg/day was suggested for pediatric MDD patients with body weight of 30–80 kg; for twice-daily sertraline scheme, the dosage of 0.5 mg/kg/day was suggested for pediatric MDD patients with body weight of 30–80 kg.

The clinical and practical consequences of optimizing sertraline dosage in pediatric patients may guide the individualized medication of sertraline for pediatric MDD patients to reduce the treatment failure of pediatric MDD patients caused by insufficient or excessive exposure to sertraline due to drug interaction, which was of great significance for the precision medicine of pediatric MDD patients. Of course, we should also note the difficulties of applying MIPD in clinical settings. First of all, this study was based on sparse sampling sites in the real world and limited data. Therefore, in the following studies, we would carry out a prospective study with an expanded sample size. Secondly, the pediatric MDD patients were mainly in China and caution should be exercised when extrapolating children from other regions. Therefore, pediatric patients from other regions would be explored in our upcoming studies in the future. Another limitation was that we had explored all the information we could currently observe about the combination of drug and entered into a comprehensive DDI analysis, but there were still other potential drug use in pediatric MDD patients complicating other diseases, so the constantly updated potential combination of drug would be the direction of our research in subsequent investigation. Finally, the number of pediatric MDD patients taking zopiclone at the same time was a small sample size, and this was subject to clinical objective conditions, which was exactly the clinical real world drug use. In the future, we would further collect more patients taking zopiclone to verify our conclusion.

## 5 Conclusion

This study first explored the initial dosage optimization of sertraline in pediatric MDD patients based on MIPD, and recommended the optimal sertraline initial dosage in pediatric MDD patients base on zopiclone co-administration, providing basis for individualized drug administration of sertraline in pediatric MDD patients.

## Data Availability

The original contributions presented in the study are included in the article/Supplementary Material, further inquiries can be directed to the corresponding authors.
