# Supplementary material for: The effect of zopiclone co-administration on sertraline initial dosage optimization in pediatric major depressive disorder patients based on model-informed precision dosing
Source: Front Pharmacol. 2025 Jan 7;15:1470865. doi: 10.3389/fphar.2024.1470865 (PMC11747706; doi:10.3389/fphar.2024.1470865)
Supplement: Supplementary file 1 [file Table1.docx]

**Table S1. Drug interaction evaluation process of sertraline in pediatric major** **depressive disorder patients.**

| **Forward inclusion** | | | | | | | | |
| --- | --- | --- | --- | --- | --- | --- | --- | --- |
| Model number | Instructions | | | OFV | | Change of OFV | | *P* value |
| 1 | Base model | | | 1104.2 | | / | | / |
| 2 | Effect of alprazolam tablet on CL | | | 1104.06 | | -0.14 | | >0.05 |
| 3 | Effect of aripiprazole tablet on CL | | | 1102.66 | | -1.54 | | >0.05 |
| 4 | Effect of benzoxol hydrochloride tablet on CL | | | 1104.2 | | 0 | | >0.05 |
| 5 | Effect of buspirone hydrochloride tablet on CL | | | 1104.18 | | -0.02 | | >0.05 |
| 6 | Effect of clonazepam tablet on CL | | | 1100.49 | | -3.71 | | >0.05 |
| 7 | Effect of diazepam injection on CL | | | 1104.15 | | -0.05 | | >0.05 |
| 8 | Effect of haloperidol injection on CL | | | 1104.15 | | -0.05 | | >0.05 |
| 9 | Effect of levetiracetam tablet on CL | | | 1104.2 | | 0 | | >0.05 |
| 10 | Effect of lithium carbonate extended-release tablet on CL | | | 1100.85 | | -3.35 | | >0.05 |
| 11 | Effect of lorazepam tablet on CL | | | 1104.17 | | -0.03 | | >0.05 |
| 12 | Effect of olanzapine tablet on CL | | | 1103.29 | | -0.91 | | >0.05 |
| 13 | Effect of oxazepam tablet on CL | | | 1103.07 | | -1.13 | | >0.05 |
| 14 | Effect of perphenazine tablet on CL | | | 1104.05 | | -0.15 | | >0.05 |
| 15 | Effect of piropilone hydrochloride tablet on CL | | | 1103 | | -1.2 | | >0.05 |
| 16 | Effect of pregabalin capsule on CL | | | 1103.78 | | -0.42 | | >0.05 |
| 17 | Effect of propranolol hydrochloride tablet on CL | | | 1100.86 | | -3.34 | | >0.05 |
| 18 | Effect of quetiapine fumarate tablet on CL | | | 1102.55 | | -1.65 | | >0.05 |
| 19 | Effect of sodium valproate sustained-release tablet on CL | | | 1104.2 | | 0 | | >0.05 |
| 20 | Effect of sodium valproate tablet on CL | | | 1103.88 | | -0.32 | | >0.05 |
| 21 | Effect of zopiclone tablet on CL | | | 1094.36 | | -9.84 | | <0.05 |
| **Backward elimination** | | | | | | | | |
| Model number | | Instructions | OFV | | Change of OFV | | *P* value | |
| 21 | | Effect of zopiclone tablet on CL | 1094.36 | | 0 | | / | |
| 22 | | Model 21-Effect of zopiclone tablet on CL | 1104.2 | | 9.84 | | <0.01 | |

CL, clearance rate; OFV, objective function value.
